# Plant stem cells in cosmetics: current trends and future directions

**DOI:** 10.4155/fsoa-2017-0026

**Published:** 2017-07-12

**Authors:** Sonia Trehan, Bozena Michniak-Kohn, Kavita Beri

**Affiliations:** 1 Center for Dermal Research, Center for Biomaterials, Rutgers – The State University of New Jersey, Piscataway, NJ 08854, USA; 2 Department of Pharmaceutics, Ernest Mario School of Pharmacy, Rutgers – The State University Of New Jersey, Piscataway, NJ 08854, USA

**Keywords:** aging skin, antiaging, Ayurveda, cosmetics, epidermal stem cells, herbs, plant stem cells, regeneration

## Abstract

Plant regeneration at the cellular and tissue level is a unique process. Similar to animals, the stem cells in plants have properties that help stimulate and regenerate plants after injury. The unique properties of plant stem cells have been a recent area of interest and focus both in developing new cosmetics and studying how these extracts/phytohormones will influence animal skin. This special report focuses on the current evidence-based trends in plant stem cell-based cosmetics and sheds light on the challenges that we need to overcome in order to see meaningful changes in human skin using topical cosmetics derived from plant stem cells.

## Lay abstract

A new wave of cosmetic ingredients containing plant stem cells and their extracts has made its way into the industry. What role do these ingredients play in affecting the aging skin? Several ancient practices such as Ayurveda have used plants as a mainstay of treatment for thousands of years. Plant stem cells could hold an interesting role if we can harness these benefits in cosmetics to create safe and effective organic topical skin care.

Plants are equipped with a robust mechanism for regeneration of their tissues under stress. Significant efforts have been put into understanding this mechanism in the expanding field of plant biotechnology [1]. In animals, tissue regeneration occurs following a wound stimulus, resulting in the initiation of organ repair. By contrast, the regenerative efforts made by the plants are not only for tissue repair from damage, but also for the development of a new plant [2]. In other words, cutting the branch of a tree also results in a new bud/branch growth. Can we use this unique property in plants for improving tissue regeneration in animals? “Eat an apple a day…But can it keep aging away?” is thus the question. This report focuses on current applications of plant stem cell-based cosmetics and current research into effects of plant stem cells in human skin.

## Aging epidermal stem cells & the concept of callus regeneration in plants

Aging of skin is a complex process involving all layers of the epidermis and dermis, comprising denaturing proteins and reduced functioning of regenerative stem cells. Stem cell location in the epidermis is divided into basal layer stem cells and interfollicular stem cells [3]. A decline in the function of epidermal stem cells has been observed in association with shortened telomeres, which reduced proliferative potential in response to UV exposure leading to premature skin aging and senescence [4]. Telomeres are nucleoprotein complexes that cap and save the ends of chromosomes from degradation and abnormal recombination [4]. Telomeres shorten with each cell division and progressive telomere shortening ultimately results in cellular senescence. The question therefore is “Can plant phytohormones have the antioxidant and regenerative ability to prevent this aging process in human skin?”

The phenomenon of callus creation from differentiated adult plant cells was described for the first time in 1902 by the Austrian botanist, Gottlieb Haberlandt [5]. He suggested that the individual plant cell is able to regenerate the entire plant. This itself was demonstrated in 1958 by cloning a carrot from *in vitro* cultivated carrot cells [6]. Since then, many articles have been published dedicated to regeneration of the entire plant from the cultivated cells and/or tissues. The callus creation process is one stage of somatic embryogenesis (i.e., formation of a zygote without fertilization) and the plant cells are subjected to dedifferentiation to again become stem cells capable of producing a new tissue or even an entire organ. The *WUS* protein is responsible for turning somatic cells back into stem cells. Research shows that cytokines are responsible for the production of stems from a callus, while auxins are responsible for the production of roots [6]. There is evidence that shows plant auxins have a regulatory effect on telomere length [7].

## Current trends in plant-based cosmetics

Animals uphold a stem cell pool as mother cells with the capability of continuous differentiation into any type of more specialized cells for various tissues in the body, such as heart muscles, skin tissues and liver tissues. However, in plants this process is more adaptable to establish stem cell niches in new locations [8]. A significant challenge for the cosmetic industry is to provide functional, ‘Â trendy’, innovative and safe products with a longer shelf life. Due to the objectionable use of human or animal sources, all cosmetic research and development for new products is consequently focused on biotechnology and plant cell culture technology to overcome the industrial, consumer and legislative constraints. Plants of cosmetic interest have been limited for use due to slow growth, seasonal harvest, variation of active concentration from plant to plant and harvest to harvest and existence of toxic metabolites.

Plant cell culture techniques have been shown to overcome these crucial problems in manufacturing cosmetic products, with the benefit of higher production of active concentrations through stimulating factors such as UV radiation, jasmonic acid or toxic substances [9]. This technology involves many complex methods that ensure growth of plant cells, tissues or organs in the environment with microbe-free nutrients. This technology allows synthesis of biologically active substances that exist in plants but that are either not usually available in the natural environment or are difficult to obtain by chemical synthesis. The extracts obtained through this technology from plant stem cells are currently used for production of both regular consumer or professional care cosmetics, such as whitening agent, arbutin obtained from *Catharanthus roseus* (rose periwinkle) and pigments such as safflower and saflorin from *C. tincorius* [10]. The various steps in the extraction process involve selection of suitable plant material and its sterilization, callus induction and subcultivation on commercially available Murashige and Skoog plant tissue culture medium. The selection of the appropriate cell line can be based on the highest biomass production and shortest doubling time. Established suspension culture can be processed with high pressure homogenization to break the suspended cells completely and entirely release the active ingredients. Thus, produced extract of plant stem cells can be encapsulated in various carrier systems for better topical delivery as a cosmetic product. Research on the use of plant stem cells as skin care is still in its infancy. Some research in the past few years has demonstrated plant cell culture technology as an effective method for extraction of stem cells in the development of novel cosmetic plant derived actives [10]. As an example, Schmid *et al*. produced stem cells of an old rare apple plant grown in Switzerland with very good storage properties, by applying plant cell culture technique. The extract of the cultured apple stem cells was obtained after plant cells lysis using high pressure homogenization and had shown multiple beneficial applications [11].

The company Mibelle AG Biochemistry at Buchs (Switzerland) carried out experiments where they incubated human fibroblasts induced with typical aging symptoms of damage to cellular DNA, in 2% stem cell extract from Uttwiler Spätlauber that could reverse the aging process of skin fibroblasts by upregulating the expression of several genes vital for cellular proliferation and growth as well as stimulate the expression of the valid antioxidant enzyme hemeoxygenase-1 [11]. It also demonstrated effectiveness in enhancing the viability of umbilical cord blood stem cells and to increase the lifespan of isolated human hair follicles. Lecithin liposomes were used as a delivery carrier for the extract. Clinical trial of *Malus domestica* (Phytocell Tech™), a liposome encapsulated extract of cultured apple stem cells as a cosmetic ingredient has shown significant potential to reduce wrinkles in the crow's feet area of the face [11]. Depth of wrinkles was measured using the optical device, PRIMOS system for 3D skin surface display, and showed that the wrinkles became shallower by 8% after 2 weeks and shallower by 15% after 4 weeks.

Another research group [12] developed an efficient production process of cloudberry (*Rubus chamaemorus*) cells in bioreactors (from 2.5 to 300 l mass production culture) from established callus and suspension cultures of the Nordic wild berry plant, cloudberry, using Murashige and Skoog medium fortified with the phytohormones kinetin and α-naphthalene acetic acid. The produced cloudberry cell material could be utilized as raw material to be used in the cosmetic industry. The established process could be exploited for fresh cells or cell fractions, extracts and isolated compounds possessing potential bioactivities, freeze-dried cell material, fragrance or color as a sustainable production technology for other plant species.

Tomato cultured stem cells from the plant *Lycopersicon esculentum* have shown significant potential in protecting skin from heavy metal toxicity [13]. A hydrosoluble cosmetic active was produced from liquid cultures of *L. esculentum* with much higher concentrations of some flavonoids and phenolic acids such as rutin, coumaric, protocatechuic and chlorogenic acids. The tomato stem cell extract contained a high content of antioxidants and metal-chelating compound phytochelatins, which capture metals and prevent the damage of the cellular structures, and suggest other remarkable applications in skin care cosmetic formulations to support healthy skin [14].

Refined ginger consists of active plant cells made from the medicinal Asian ginger plant using a specific biotechnology mixing plant cell dedifferentiation and a plant cell culture controlling the synthesis of active molecules inside cells. A clinical study, as reported by the manufacturer, performed on 22 women indicated an improvement of around 50% in skin structure by pore reduction and a mattifying effect revealed by a reduction of shininess by 15% after 6 h and a reduction of sebum by 19% after 28 days. *In vitro* test results have shown an increase in the synthesis of elastin and fibers and reduced sebum production rate [15].

An Italian biotechnology company, the Institute of Biotechnological Research (now a part of Croda via the Sederma brand) has explored the protective effect and strong anticollagenase and hyaluronidase activity of an antiaging ingredient from edelweiss stem cells. *Leontopodium alpinum* has high concentrations of leontopodic acids A and B, which have strong antioxidant properties. They have used patented High Tech Nature technology [16] ensuring reproducible composition and active ingredient titer to produce ingredients for industrial quantities [17].

XtemCell's patented stem cell technology uses active plant cells from rare, 100% organic, nutrient-rich plants to create new cells high in purity and nutrients rather than using the traditional technique utilizing harsh chemicals for extraction. This patented technology ensures high concentrations of lipids, proteins, amino acids and phytoalexins [18]. According to the company, clinical testing has proven that the active cells used in XtemCell's products are easily absorbed into the outermost cells of the epidermis, allowing almost instantaneous skin cell renewal, nutrient absorption and an increase in the skin's level of filaggrin proteins to protect the skin from further sun and aging damage when applied [19,20].

## Plant stem cells versus plant stem cell extracts

Terminology is crucial in claims made by cosmeceuticals, for example, understanding that when the term ‘plant stem cell’ is used as an ingredient, it actually refers to the extract of the primitive cell.

Many skin care companies are promoting their products with the claim of utilizing stem cell technology [21]. For example, Image Skincare have a series of products such as antiaging serum, lightening cream, lightening cleanser and lotion [21].

Furthermore, stem cell products such as Dermaquest Stem cell 3D HydraFirm serum, Peptide eye firming serum are advertised to contain gardenia, echinacea, lilac and orange stem cells.

In fact, almost all cosmetic companies advertising to contain stem cells in their products actually contain stem cell extracts and not the live stem cells.

Although research on plant stem cells used in skin care reveals their potential as skin protectives, antiaging and antiwrinkle products, the actual stem cells in cosmetic formulations are already dead. Extracts from stem cells cannot act in the same way as the live stem cells. Claimed benefits of smooth and firm skin are due to antioxidants and active extracts from stem cells. To gain all the authentic benefits from stem cells and to let them work the way they are promised to in skin care applications, they need to be incorporated as live cells and should remain so while in the cosmetic formulation. Incorporating stem cells in a carrier which can assist the cells to penetrate deep into the skin to provide an actual cosmetic benefit is another challenge to be addressed. Plant stem cell therapy needs to move in the right direction to implement its inherent potential in skin care. This might happen in the next 20 years but any cosmetic that is advertised to be antiaging due to plant stem cells at this time is about as effective as all the skin creams without stem cells.

## Conclusion & future perspective for plant stem cells in cosmetics

Plants have a complex phytohormone cascade that regulates plant growth and regeneration. There is yet much to understand as to how phytohormones control the regeneration process and if this can be extrapolated to regeneration in human tissues. The active ingredients in the phytohormone cascade that have actions on human stem cells and human tissues are areas of interest and focus for the future of plant stem cell research [22]. It could be considered that: ‘an apple a day can keep aging skin away and perhaps help regenerate skin’. The authors’ goal in writing this article was to promote additional research on plant stem cell biotechnology and its effect on human skin and improve current cosmetics with safer and effective organic ingredients. Ancient herbal sciences such as Ayurveda and Oriental Chinese Medicine have used plants as their mainstay for the treatment of chronic ailments, acute inflammation and for healing. In current times, for innovative cosmetic scientists, the goal is to connect the evidence from ancient practices to evidence in modern science and see where the application of plants in cosmetics can improve the delivery to the skin in a more effective, safer and targeted way. Plant science in this vein is currently in its infancy and research in herbal biotechnology and physiologic effects on the skin could open new doors in cosmetics. An interesting aspect for future research will be the discovery of actives of phytohormones that act directly or influence repair pathways for human tissues. It is possible in the future to combine and expand our understanding of the basis of Ayurvedic and ancient medicine with its use of plant herbs to shed more light on cutting-edge scientific research.

**Executive summary**

- The regenerative properties of plants has grabbed the interest of dermatology researchers and the cosmetics industry.
- The restrictions on the use of animals and humans in cosmetics has also turned interest toward plants.
- Plant-based cosmetics have their own disadvantages, such as difficulties in cell culture. New technologies are aiming to overcome these, yet the research field remains in its infancy.
- While many cosmetics currently claim to utilize plant stem cells, they in fact use plant stem cell extracts.
- Live cells are likely to be more advantageous, which raises a need for research in delivery methods.
- Additional research into plant stem cells and the associated biotechnology is clearly required to improve current cosmetics.
